# Daily serum phosphate increase as early and reliable indicator of kidney injury in children with leukemia and lymphoma developing tumor lysis syndrome

**DOI:** 10.1007/s00467-023-05923-z

**Published:** 2023-03-21

**Authors:** Erika Biró, Dániel Erdélyi, Petra Varga, Mária Sinkó, Katalin Bartyik, Gábor Kovács, Gábor Ottóffy, Ferenc Vincze, István Szegedi, Csongor Kiss, Tamás Szabó

**Affiliations:** 1 Division of Nephrology, Department of Pediatrics, Faculty of Medicine, University of Debrecen, 98 Nagyerdei Krt, Debrecen, 4032 Hungary; 2 2nd Department of Pediatrics, Faculty of Medicine, Semmelweis University, 7-9 Tűzoltó U, Budapest, 1094 Hungary; 3 Department of Pediatrics, Albert Szent-Györgyi Health Centre and University, 14-15 Korányi Fasor, Szeged, Hungary 6725; 4 Department of Pediatrics, Medical School, University of Pécs, 7. József Attila U, Pécs, 7623 Hungary; 5 Department of Public Health and Epidemiology, Faculty of Medicine, University of Debrecen, 1. Egyetem Tér, Debrecen, 4032 Hungary; 6 Division of Pediatric Haematology-Oncology, Department of Pediatrics, Faculty of Medicine, University of Debrecen, 98 Nagyerdei Krt, Debrecen, 4028 Hungary

**Keywords:** Phosphate, Uric acid, Acute kidney injury, Tumor lysis syndrome, Kidney replacement therapy

## Abstract

**Background:**

Tumor lysis syndrome (TLS) and its most serious complication, acute kidney injury (AKI) are one of the emergency conditions in onco-hematology. It is difficult to predict the degree of kidney involvement. Therefore, we studied children with leukemia and lymphoma treated in four Hungarian tertiary centers (inpatient university clinics) retrospectively (2006–2016) from a nephrological aspect.

**Method:**

Data of 31 pediatric patients were obtained from electronic- and paper-based medical records. Physical status, laboratory test results, treatments, and outcomes were assessed. Patients were analyzed according to both “traditional” TLS groupings, as laboratory TLS or clinical TLS, and nephrological aspect based on pRIFLE classification, as mild or severe AKI.

**Results:**

Significant differences were found between the changes in parameters of phosphate homeostasis and urea levels in both classifications. Compared to age-specific normal phosphate ranges, before the development of TLS, hypophosphatemia was common (19/31 cases), while in the post-TLS period, hyperphosphatemia was observed (26/31 cases) most frequently. The rate of daily change in serum phosphate level was significant in the nephrological subgroups, but peaks of serum phosphate level show only a moderate increase. The calculated cut-off value of daily serum phosphate level increased before AKI was 0.32 mmol/L per ROC analysis for severe TLS–AKI. The 24-h urinalysis data of eight patients revealed transiently increased phosphate excretion only in those patients with TLS in whom serum phosphate was elevated in parallel.

**Conclusion:**

Daily serum phosphate level increase can serve as a prognostic factor for the severity of pediatric TLS, as well as predict the severity of kidney involvement.

**Graphical abstract:**

**Figure:**
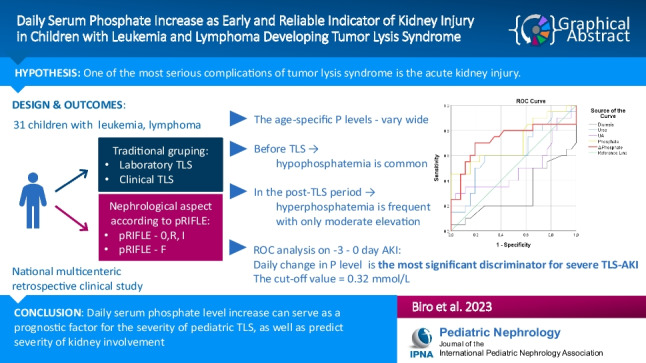
A higher resolution version of the Graphical abstract is available as Supplementary information

**Supplementary Information:**

The online version contains supplementary material available at 10.1007/s00467-023-05923-z.

## Introduction

Tumor lysis syndrome (TLS) is characterized by hyperuricemia, hyperphosphatemia, hyperkalemia, and hypocalcemia [1]. According to the Howard modification of the Cairo–Bishop criteria, at least two of the abovementioned parameters exceed the normal ranges with a margin of 25% at the same time (in 24 h) [2, 3].

Clinically significant TLS (CTLS) is known to be associated with high morbidity and mortality, as its rapid progression may result in severe organ damage including kidney impairment, seizures, cardiac arrhythmias, pulmonary edema, or even death [3, 4]. Acute kidney injury (AKI) is one of the most common complications and an important predictor of short- and long-term mortality [5].

The pathomechanism of TLS is complex, consisting of both crystal-dependent nephropathy and certain crystal-independent mechanisms, which can cause endothelial damage and microvascular dysfunction [6, 7]. Glomerular filtration rate (GFR) and renal blood flow were frequently found reduced by approximately 50% during kidney examination already in cases with mild hyperuricemia, which may indicate early kidney involvement [8–10].

The current TLS management of the Hungarian Pediatric Oncology Group (HPOG) corresponded with the guideline of the “British Hematology Standards Committee,” in which patients were grouped on the basis of risk classification [11, 12]. In “low-risk” patients the guideline-recommended aggressive hydration with the use of allopurinol and a “watch and wait” strategy, even though this approach might only decelerate the TLS-related pathological processes [13]. One of the main elements of conservative treatment in the “intermediate” and “high” risk TLS patients is the use of recombinant urate-oxidase (Rasburicase®) (rUO), which is capable of removing uric acid (UA) effectively [14, 15]. However, the influence of rUO treatment and UA levels achieved by rUO treatment is controversial in terms of developing kidney failure. Several studies have come to result that the effect of rUO treatment on risk for AKI was not significant according to multivariate modeling [16, 17]. The use of rUO early in the course of AKI may mitigate further kidney damage, especially in mild AKI, where regeneration is also faster with the application of rUO, but the outcome of severe AKI may not be affected significantly [14, 18]. The pediatric dose of rUO treatment also differs in the literature due to its high cost and severe side effect profile. There was no significant difference between the fixed dose and doses optimized for body weight in terms of the incidence of AKI [19].

In severe cases, the treatment should be combined with kidney replacement therapy (KRT) [11, 20], but there is no consensus in the modality and timing of KRT [21, 22]. Early introduction of KRT has been proven to be kidney protective with better long-term kidney survival [20, 22]. In comparative studies of early and late initiation of dialysis, the increase in the frequency of cannula sepsis was highlighted as a major disadvantage [22, 23].

Recently, in parallel with the increasing use of highly effective anti-cancer treatments, the incidence of TLS has been growing [4, 14, 24]. Early recognition of AKI in TLS is important in guiding further management of these patients.

The objective of our multicenter study was to perform a comprehensive analysis of patients with TLS from a nephrological perspective. We analyzed the incidence and characteristics of AKI among pediatric patients with TLS on the basis of clinical and laboratory data, including kidney function tests, electrolyte levels, and 24-h urine samples. We aimed to select the best-performing conventional biomarker for the prognosis and severity of pediatric TLS and predict the development of AKI.

## Material and methods

### Study design

This retrospective clinical investigation was carried out between 2006 and 2016 and included children with leukemia and non-Hodgkin lymphoma (NHL) with at least two laboratory abnormalities characteristic of TLS according to the Cairo–Bishop criteria [2, 15]. The study was performed in tertiary pediatric hematology-oncology divisions at four university hospitals (Semmelweis University, Budapest; University of Debrecen, Debrecen; University of Pécs, Pécs; and University of Szeged, Szeged) in Hungary. Data were obtained from the patients’ paper- and electronic-based medical documentation and from the database of the Hungarian Pediatric Tumor Registry.

### Patients

**Fig. 1:**
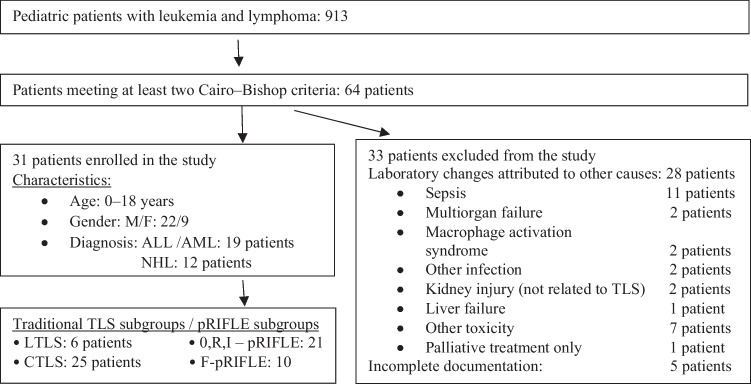
Patient enrolment. Flow-chart of patient selection and clinical characteristics of patients

All children with leukemia and lymphoma, who were treated in the above centers during the study period (01.01.2006–12.31.2016) were included in our investigation, a total of 913 children. Sixty-four patients were selected based on predefined laboratory criteria of TLS. Twenty-eight children were excluded from further analysis as laboratory changes were attributed to potential causes different from TLS, and an additional 5 further patients were excluded because of incomplete documentation (Fig. 1).

The remaining 31 patients were categorized according to two classifications, i.e., “traditional” TLS (Laboratory TLS (LTLS) and CTLS) and nephrological point of view based on the calculated GFR values and urine volume using the pRIFLE classification. In the grouping, we distinguished between no and mild kidney damage (0,R,I-pRIFLE) and severe kidney damage (F-pRIFLE) (Fig. 1.)

Patients’ characteristics were further analyzed on the basis of the general condition of patients, their medication, laboratory findings, and applied clinical interventions. The nephrotoxic drug burden of patients was determined according to the publication by Ehrmann et al. [25]. This calculation was applied retrospectively in all patients for the 5 days preceding the development of TLS.

Treatment of patients with TLS–AKI was performed according to international recommendations, also accepted by HPOG, using a multidisciplinary approach [11, 26]. The university treatment protocols were the same in terms of hydration and allopurinol use, as well as KRT indication.

As the examination period was relatively long, the treatment of patients included was not completely uniform in terms of rUO use. Over the 10 years of the study period, about 50% of the patients received rUO.

Clinical and laboratory characteristics and treatments of patients are included in Table 1.

**Table 1:** Clinical and laboratory characteristics of patients

Groups	All	“Traditional” subgroup		Nephrological subgroup	
	*n* = 31	LTLS *n* = 6 (19%)	CTLS *n* = 25 (81%)	pRIFLE: 0, R, I *n* = 21 (67%)	pRIFLE: F *n* = 10 (33%)
Patients general information					
Age (years), Med (IQR)	10.5 (5.62–14.7)	4.5 (1.37–8.37)	11.5 (6–15)	6.5 (4–11.5)	15.2 (13.1–16.7) ***p* = 0.006**
Gender (male), *n* (%)	22 (70%)	3 (50%)	19 (76%)	14 (66%)	8 (80%)
Gender (female), *n* (%)	9 (30%)	3 (50%)	6 (40%)	7 (34%)	2 (20%)
Patients laboratory selection criteria according to Cairo–Bishop pediatric criteria, *n* (%)					
Phosphate ≥ 2.1 mmol/L	28 (90%)	5 (83%)	23 (92%)	19 (90%)	9 (90%)
UA ≥ 476 umol/L	21 (67%)	6 (100%)	15 (60%)	16 (76%)	5 (50%)
Ca ≤ 1.75 mmol/L	19 (61%)	2 (33.2%)	17 (68%)	11(52%)	8 (80%)
K ≥ 6.0 mmol/L	6 (19%)	1 (16.6%)	5 (20%)	3 (14%)	3 (30%)
Characteristics of TLS-related clinical symptoms, *n* (%)					
Clinical symptoms of TLS: • Total:	17 (54%)	–	17 (68%) ***p* = 0.02**	5 (23%)	12 (120%) ***p* < 0.001**
• Tetany due to hypocalcemia	8 (25%)	–	8 (32%)	4 (19%)	4 (40%)
• Arrhythmia	1 (3.2%)	–	1 (4%) ***p* = 0.032**	1 (5%)	–
• Oliguria presence	8 (25%)	–	8 (32%)	–	8 (80%) ***p* < 0.001**
Characterization of AKI					
• AKI according to CTLS definition/Traditional Classification, *n* (%): Se Cr > 1.5 × upper normal limit of patient age and sex	25 (80%)	–	25 (60%) ***p* < 0.001**	15 (75%)	10 (46%)
pRIFLE subgroups, *n* (%)			***p* < 0.001**		
• no AKI	4 (13%)	4 (67%)	–	4 (19%)	–
• pRIFLE R	2 (7%)	2 (33%)	–	2 (10%)	–
• pRIFLE: I	15 (48%)	–	15 (60%)	15 (71%)	–
• pRIFLE: F	10 (32%)	–	10 (40%)	–	10 (100%)
Diuresis (ml/kg/h), Med (IQR)	3 (1.57–3.75)	3 (3–3.37)	3 (1.2–4) ***p* < 0.001**	3.2 (3–4)	1.13 (0.9–1.4) ***p* < 0.001**
Dialysis, *n* (%)	9 (29%)	–	9 (36%)	–	9 (90%) ***p* < 0.001**
General parameters of TLS patients, *n* (%)					
Preexisting abnormality					
• Cardial	3 (9.6%)	–	3 (12%)	1 (4.7%)	2 (20%)
• Nephrological	2 (0.06%)	–	2 (8%)	1 (4.7%)	1 (10%)
TLS type:					
• Spontaneous	9 (29%)	3 (50%)	6 (24%)	6 (28%)	3 (30%)
• After chemotherapy initiation	22 (71%)	3 (50%)	19 (76%)	15 (72%)	7 (70%)
Acute leukemia	19 (61.2%)	6 (100%)	13 (52%)	14(66%)	5 (50%)
• ALL	18 (64%)	6 (100%)	12 (92%)	14 (100%)	4 (80%)
• AML	1 (6%)	–	1 (8%)	–	1 (20%)
Non-Hodkin lymphoma	12 (38%)	–	12 (48%)	7 (34%)	5 (50%)
• NHL, non-Burkitt Ly.	2 (18%)	–	2 (16.6%)	1 (14%)	1 (20%)
• Burkitt lymphoma	10 (82%)	–	10 (83.4%)	6 (86%)	4 (80%)
Patient risk stratification, *n* (%)			***p* = 0.016**		
• “Intermediate” risk	4 (12.9%)	3 (50%)	1 (4%)	3 (14.3%)	1 (10%)
• “High” risk	27 (87.1%)	3 (50%)	24 (96%)	18 (85.7%)	9 (90%)
Main laboratory parameters of patients					
Highest serum UA level (mmol/L), Med (IQR)	600 (478–910)	561 (544–688)	976 (466–976)	573 (493–767)	782 (482–1034)
∆UA /day, Med (IQR)	89 (68–119)	75 (69–123)	94 (71–116)	72 (64–178)	100 (89–105)
LDH in NHL (mmol/L), Med (IQR)	4115 (2611–4528)	–	1994 (1826–2253)	2971 (1792–3996)	4593 (4462–5509) ***p* = 0.042**
WBC in ALL (G/L), Med (IQR)	64 (15.9–308)	15.4 (8.1–42)	124 (21.8–374)	79.5 (21–333)	38 (19.6–72)
DIC, *n* (%)	3 (9%)	–	3 (12%)	2 (9.5%)	1 (10%)
Serum K level at the TLS onset (mmol/L), Med (IQR)	4.3 (4–4.6)	4.4 (4.07–4.7)	4.3 (4–4.6)	4.25 (3.95–4.65)	4.4 (4.0–5.5)
Serum K level at the lowest GFR(mmol/L), Med (IQR)	4.4 (3.8–5.3)	4.4 (3.85–4.4)	4.3 (3.7–5.3)	4.35 (3.85–4.85)	4.9 (4–6)
Serum urea level at the onset of TLS (mmol/L), Med (IQR)	7.9 (6–23.4)	5.3 (4.5–5.72)	13.5 (7.4–26.1) ***p* = 0.031**	6.5 (4.3–8.6)	24.9 (18.9–34.9) ***p* < 0.01**
Serum urea level at the lowest GFR (mmol/L), Med (IQR)	22.5 (8.2–32.5)	5.3 (5.1–6.1)	25.3 (18.5–35.8) ***p* = 0.007**	7.11 (5.8–25.3)	32.1 (22.7–38.07) ***p* < 0.01**
Phosphate homeostasis with the use of age-related normal values					
Hypophosphatemia before TLS (− 3 to − 1 day), *n* (%)	19 (61%)	3 (50%)	16 (64%)	11 (52%)	8 (80%)
Max. degree (%) in decreases of phosphate level, Med (IQR)	24,4 (15.3–47.7)	41.7 (16.6–54.8)	26 (12.6–47.7)	21.5 (14–41.9)	36 (20–47)
Min. Phosphate level (mmol/L), Med (IQR)	1.03 (0.72–1.27)	1.1 (0.88–1.34)	1.03 (0.73–1.2)	1.18 (0.97–1.3)	0.75 (0.72–1.2)
Hyperphosphatemia, *n* (%)	26 (83%)	4 (66%)	22 (88%)	17 (80%)	9 (90%)
Max. degree (%) of increment in phosphate level, Med (IQR)	68.5 (37–101)	32,4 (24.5–39.5)	84 (41.6–130) ***p* = 0.033**	41.9 (35–79)	138 (90–257)
Peak of phosphate level (mmol/L), Med (IQR)	2.55 (2.19–3.35)	2.2 (2.12–2.38)	2.79 (2.23–3.85) ***p* = 0.017**	2.34 (2.19–2.9)	4 (2.87–5.69) ***p* = 0.006**
Phosphate kinetic (total change/day), Med (IQR)	0.41 (0.2–0.74)	0.35 (0.15–0.46)	0.48 (0.24–0.78)	0.45 (0.36–0.77)	0.7 (0.39–1.05) ***p* = 0.025**
Phosphate level at the lowest GFR (mmol/L), Med (IQR)	2.24 (2.01–2.7)	2.1 (2.04–2.17)	2.36 (2.01–2.72) ***p* < 0.001**	2.1 (1.99–2.38)	3.8 (2.54–4.7)***p* = 0.001**
Treatment					
Nephrotoxic drug burden, Med (IQR)	4 (0.25–6.5)	0.5 (0–3.25)	5 (1–7) ***p* = 0.046**	4 (0–6)	6.5 (1.75–10.25) ***p* = 0.08**
Inotrope score, Med (IQR)	0	0	0 (0–2)	–	2 (0.5–6.5) ***p* < 0.001**
rUO treatment, *n* (%):	16 (51%)	2 (33%)	14 (56%)	11 (52%)	5 (50%)
• (ALL	(8	(2	(6	(5	(3
• AML	1	–	1	–	1
• NHL, non-Burkitt Ly	1	–	1	1	–
• Burkitt Ly.)	6)	–)	6)	5)	1)
Mechanical ventilation required, *n* (%)	6 (19%)	–	6 (24%)	3 (14.2%)	3 (30%)
Leukapheresis, *n* (%)	2 (6%)	–	2 (8%)	1 (4.7%)	1 (10%)
Pleural, pericardial drain, *n* (%)	3 (9.6%)	–	3 (12%)	1 (4.7%)	2 (20%)
Intestinal resection, *n* (%)	1 (3.2%)	–	1 (4%)	1 (4.7%)	–
Resuscitation, *n* (%)	1 (3.2%)	–	1 (4%)	–	1 (10%)

General information and main laboratory findings of patients are shown. The patients are selected according to the “traditional” TLS grouping and the severity of kidney injury according to pRIFLE criteria: mild (pRIFLE: 0, R, I)/severe (pRIFLE: F) kidney injury. Data are expressed as median (Med) with interquartile range (IQR) or patient number (*n*) with percentile (%). According to the Mann–Whitney and Fisher exact analysis of these data, the significant differences are indicated by specifying the *p* value

### Nephrological assessment

The GFR was calculated according to Bedside Schwarz Eq. 2009 formula [27]. Assessment of changes in serum and urine phosphate levels was based on age-specific normal serum phosphate ranges and 24-h collected urine findings.

The age-specific normal serum phosphate range [28]:

**Table:** 

Age (years)	0–0.5	0.5–1	1–5	6–12	13–20
Serum phosphate (mg/dL)	5.2–8.4	5–7.8	4.5–6.5	3.6–5.8	2.3–4.5

Data of 24-h urine examination were available in 8/31 patients. Urine parameters and their formulas are as follows [29–31]:

- u(urine) Phosphate/Cr (creatinine) = u Phosphate/uCr ratio (mmol/mmol)
- The ratio of the maximum rate of renal tubular reabsorption of phosphate to GFR:TmPO4/GFR = serum (se) Phosphate – (u Phosphate/uCr) ×seCr
- Fractional excretion (FE) of Phosphate% = [u Phosphate (mg/dL)/se Phosphate (mg/dL)] × [seCr (mg/dL)/uCr (mg/dL)] × 100
- Ca (calcium) /Cr ratio (mmol/mmol) = [24-h uCa × seCr]/[seCa × 24-h uCr]
- Ca excretion (mg/kg/24 h)
- FE of Sodium (Na) (FENa)% = 100 × (seCr (mg/dL) × uNa)/(seNa × uCr (mg/dL)

### Statistical analyses

Statistical analyses were conducted using SPSS 24.0 (IBM Corp. Released 2016. IBM SPSS Statistics for Windows, Version 24.0. Armonk, NY: IBM Corp.). Categorical values are presented as case numbers (*n*) and percentages (%), and continuous data are expressed as median values (Med) with the corresponding interquartile range (IQR). We compared the background characteristics of the subgroups with the use of univariate analyses using Mann–Whitney and Fisher exact tests, taking into account the low sample size and non-normality of the data.

We investigated the discriminatory ability of clinically important laboratory markers for TLS by using the ROC analysis for the severe TLS–AKI (pRIFLE: F) subgroup and checked the cut-off values.

## Results

### Patient characteristics

The incidence of TLS in our study was 31/913 patients (3.4%). We observed nine patients with spontaneous TLS. There were 6/31 LTLS (19%) and 25/31 CTLS (80%). In terms of gender, a male predominance was observed, and the median age increased in parallel with the severity of TLS. According to the Cairo–Bishop criteria, hyperphosphatemia was present in 28 patients (28/31), followed by hyperuricemia (21/31), hypocalcemia (19/31), and hyperkalemia (6/31).

The most common laboratory anomaly associated with TLS was elevated serum Creatinine indicating kidney injury (25/31), based on the definition of CTLS. Nine of 25 patients (36%) required KRT due to hyperphosphatemia (7/9) and oliguria (2/9).

The LTLS group mostly consisted of patients with mild kidney impairment (pRIFLE: 0, R); the CTLS group comprised patients with moderate AKI (pRIFLE: I) and severe (pRIFLE: F) AKI. There was only one patient who did not meet the criteria of KRT but had severe AKI (pRIFLE: F).

Based on the statistical analysis of the “traditional” classification, we found a significant difference in the total number of clinical symptoms of TLS, as well as the distribution of patients in the risk groups and under a nephrotoxic drug burden. Among the main laboratory parameters some, such as the serum levels of urea (both at the onset of TLS and during kidney failure) and some parameters representing phosphate homeostasis, showed significant differences.

Hypophosphatemia on days − 3 to − 1 preceding the onset of TLS was detectable altogether in 19/31 cases (61%), and in parallel with the severity of the disease, an increasingly severe initial hypophosphatemia was observed. As expected, hyperphosphatemia was detected in most patients (83%) (Table 1).

### Analysis of kidney failure

Patients were divided into two groups: the severe AKI (pRIFLE: F) group and the mild AKI (pRIFLE: 0, R, I) group, in order to analyze from the nephrological aspect.

Statistical analysis of TLS syndrome according to both groups revealed similar differences, such as TLS-associated clinical symptoms and the urea levels at the onset of TLS and at the lowest GFR. Furthermore, there were significant changes in the phosphate level at the lowest GFR and the peak phosphate level.

The importance of daily serum phosphate level increase was revealed from statistical data performed according to the nephrological classification. A significant difference in nephrotoxic drug burden and patient numbers based on risk stratification assessment, which is an important determinant of TLS management, was found only in the “traditional” grouping (Table 1).

**Fig. 2:**
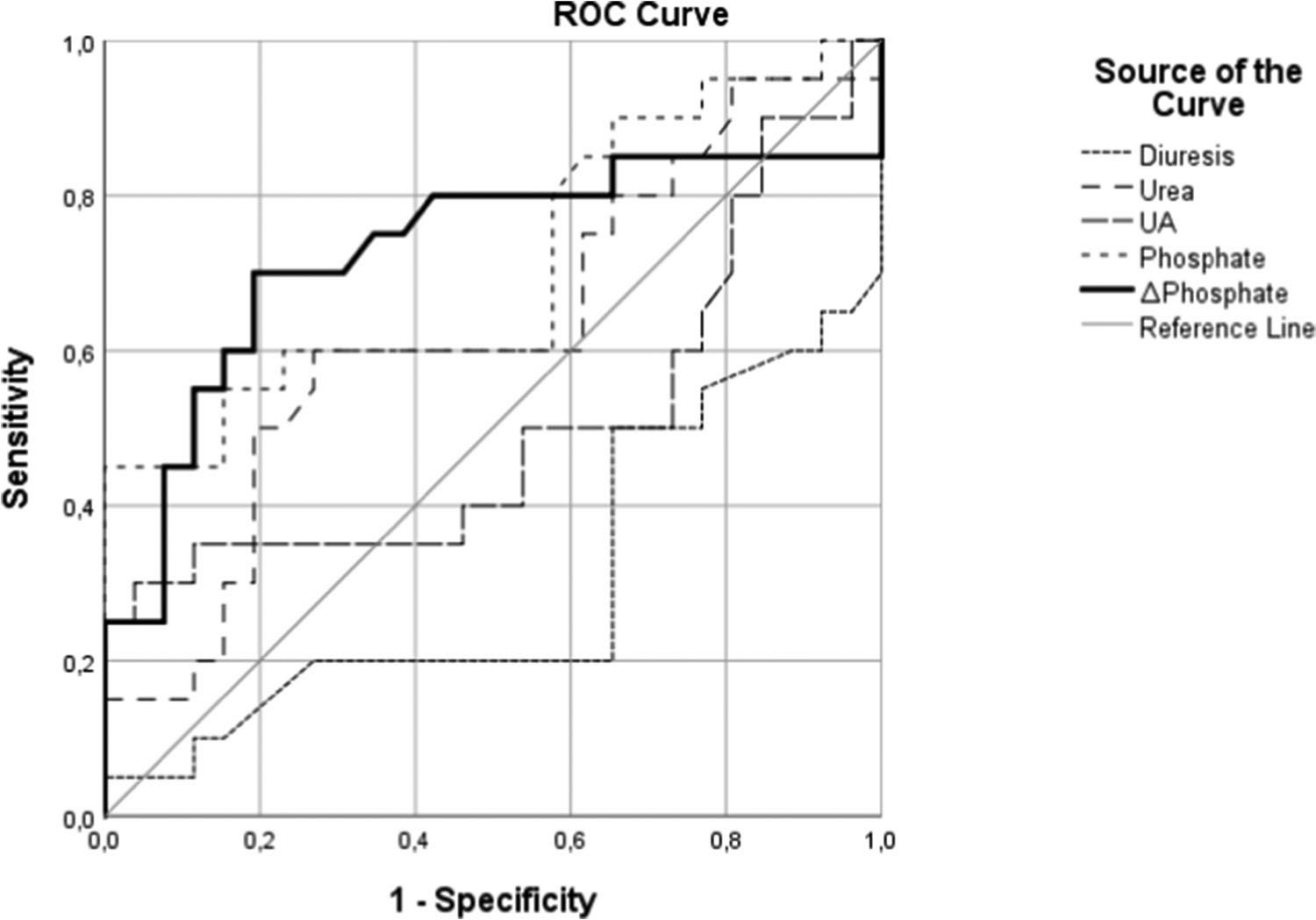
Predictors of severe AKI. Analysis of AKI (pRIFLE: F) predictors according to ROC analysis during − 3 to 0 AKI days

The daily change in serum phosphate level before the onset of AKI (− 3 to 0 days) proved to be the most significant discriminator for severe TLS–AKI, as demonstrated by ROC analysis (AUC = 0.727, *p* = 0.009). The cut-off value of daily change in serum phosphate concentration was 0.32 mmol/L. The ROC curve also shows that UA values varied in a wide range. The most accurate estimate found was the change in daily phosphate levels. A significant difference was revealed in two additional parameters, diuresis as measured in ml/kg/h and the peak of the phosphate level, with both weaker specificity and sensitivity compared to the change in daily phosphate (Fig. 2).

## Evaluation of collected urine samples

According to the 24-h collected urinalysis samples, the direction of changes in urine and serum phosphate parameters, such as TMPO4/GFR, FE of phosphate, and serum phosphate levels were similar with an intensity depending on previous kidney injury and/or tumor cell turnover. In three cases, low GFR values (< 60 ml/min/1.73 m^2^) were observed in addition to elevated FENa, FE of phosphate (in two cases each), and TMPO4/GFR (in all three cases). All these parameters normalized later. Increased urinary Ca excretion was observed in two cases (Table 2).

**Table 2:** Urinalysis

TLS Type	Patient (year)	Day from start of TLS	GFR	early Se phosphate (mmol/L) Se	Se phosphate (mmol/L)	SeUA (umol/L)	SeCa (mmol/L)	FENa (%)	FE Phosphate (%)	TMPO4/GFR	Phosphate/creatinine (mmol/mmol)	Ca excr. (mg/kg)	Se phosphate (mmol/L)
LTLS	#1 (9)	3	93	Normal	2.18 (↑)	73	2.5	2.07	8	2.18	0.31 (↓)	0.072	4.42
CTLS	#2 (4.5)	− 1	91	1.92	1.92	726	1.8	1.73	10.3	1.92	4.66	2.68 (↑)	14.1 (↑)
		2	84		1.31 (↓)	156		1.88	16.3	1.31	4.66	2.68 (↑)	14.1 (↑)
CTLS	#3 (6.5)	0	47	0.81 (↓)	2.76 (↑)	449	1.79	0.12	32.7 (↑)	2.75 (↑)	8.93 (↑)	0.35	2.17
		8	131		1.18 (↓)	–	2.49	1.16	3.8 (↓)	1.18	1.2	0.95 (↑)	2.9
CTLS	#4 (15)	− 2	54	0.5 (↓)	0.57 (↓)	283	2.5	1.33	0.44 (↓)	0.57 (↓)	0.04 (↓)	0.37	1.71
CTLS	#5 (5.5)	3	26.5	Normal	3.72 (↑)	160	2.2	12.7 (↑)	15	3.71 (↑)	3.83	1 (↑)	4.22 (↑)
CTLS	#6 (11.5)	− 3	96	1 (↓)	1.03 (↓)	319	2.42	0.42	–	0.39 (↓)	–	–	0.35
CTLS + KRT	#7 (14.5)	− 4	46	0.27 (↓)	1 (↓)	600	1.9	1	0.5 (↓)	1 (↓)	0.035 (↓)	0.075	0.89
CTLS + KRT	#8 (16)	1	20	0.57 (↓)	5.69 (↑)	727	1.78	12.1 (↑)	45 (↑)	5.66 (↑)	0.99	(↓)	(↓)
		6	64		1.55	9	1.64	1.64	8.97 (↓)	1.45	0.44 (↓)	0.53	4.42

Results of 24-h urine samples collected from eight patients. Arrows indicate the deviation from the age-specific reference ranges. In addition to GFR and time span of TLS (in days), the table shows the changes in Ca, phosphate, and Na homeostasis

## Discussion

Regarding the literature, the relative incidence of TLS shows a considerable variation ranging between 3 and 30% of patients with leukemia and NHL; spontaneous TLS is more common in pediatric patients than in adults [1, 32, 33]. Roughly, 20–40% of all TLS cases have clinical manifestations (CTLS). Among pediatric patients, the relative incidence of TLS–AKI has been reported to be between 5 and 40% [32], and this rate can increase up to 75% in malignancies with large tumor burden [33]. So, the severe form of TLS mainly manifests as kidney injury [1]. The dialysis rate is 2–4% [33], and the TLS-related mortality is approximately 1.7% among high-risk patients [34].

In our retrospective study, the incidence of TLS was low (3.4%). However, the ratio of more severe forms of TLS: CTLS vs. LTLS was relatively high. Early initiation of proper TLS prophylaxis and conservative treatment might have contributed to the fact that only a small number of patients had LTLS. The two most common laboratory abnormalities detected in our patient cohort were hyperuricemia and hyperphosphatemia according to Cairo–Bishop criteria with good agreement of literature [16, 35, 36].

In a study with adult patients, UA was the most sensitive predictor in the LTLS group according to the ROC curve, and in addition to higher UA levels, kidney failure also occurred more often [9]. However, in our analysis, the change in UA level did not show a significant difference.

Interestingly, in many cases, hypophosphatemia preceded TLS. The presence of hypophosphatemia before the introduction of chemotherapy has been shown to be a significant risk factor for TLS [16]. It was proposed that its development is most often caused by tumor progression (increased phosphate utilization by malignant cells) in combination with reduced intake, primary tubular disorder, or acquired tubular cell dysfunction [37, 38]. Low extra- and consequent intracellular phosphate levels may cause impairment of tubular reabsorption due to low intracellular ATP formation [38, 39]. The increased excretion of phosphate and a consequentially increased risk of nephrocalcinosis raised the possibility that these pathological events may play an important role in the development of severe TLS and TLS–AKI [16]. It is notable that the evaluation of collected urine samples in our study did not confirm any significant impairment of tubular phosphate excretion in patients without AKI.

The reason for the increased phosphate level in TLS is still debated—the most plausible explanation is cell disruption and consequent kidney failure. The renal excretion of phosphate is highly efficient in patients with normal kidney function; acute hyperphosphatemia usually resolves within a few hours (6–12 h) [40]. A number of studies showed that serum phosphate increased only when there was a substantial reduction in GFR (GFR < 60 mL/min/1.73 m^2^) [40].

Previous studies have highlighted the importance of changes in phosphate levels as a marker of AKI progression [40] and an indicator of clinical outcomes in critically ill patients [41]. Darmon et al. examined the importance of phosphate levels from the point of view of TLS. In the TLS risk assessment score serum phosphate value is one of the factors which determine CTLS. A 1 mmol/L increase in serum phosphate level was associated with a fivefold increase in CTLS risk [42]. An adult TLS study by Lemerle et al. suggested that increases in serum phosphate levels appear to be a good predictive factor for AKI–TLS. The warning peak value of serum phosphate was 2.1 mmol/L [43].

Due to significant deviations from normal phosphate levels in childhood [28] and the frequent occurrence of initial hypophosphatemia, we focused on the daily changes in serum phosphate levels. Our findings showed a significant discriminatory capacity by ROC analysis for severe TLS–AKI, where the cut-off value was determined to be 0.32 mmol/L in the daily change in serum phosphate level.

Numerous studies have investigated the role of nephrotoxic drugs as contributory factors to kidney damage in subclinical kidney involvement [26, 44–46]. Our study also showed that patients with severe TLS received a substantial number of nephrotoxic drugs.

With the development of biomarker-guided risk assessment [47], the nomenclature of kidney failure has broadened, and the use of tubular markers enables earlier recognition of AKI, which is important not only for optimizing treatment, but also for preventing chronic complications. This is complemented by the alert systems—one of the best-known of which is the RAI (renal angina index) [48, 49]. RAI is based on the estimation of general risk and the clinical symptoms characterized by changes in creatinine and fluid overload. Monitoring for the latter is particularly important, since these patients are excessively hydrated. The risk of overfilling is high in case of kidney involvement. The automated RAI + tubular marker (e.g. NGAL (neutrophil gelatinase-associated lipocalin)) clinical decision support programs [50] and calculators used to monitor nephrotoxic drugs are becoming more and more popular to promote quick recognition of AKI. Implementation of monitoring the increase of daily phosphate levels into the RAI system may help in the management of TLS patients, enabling early recognition of high-risk patients.

## Conclusion

Childhood TLS was retrospectively analyzed in our national study with a focus on nephrologic complications. Close monitoring of daily changes in the serum phosphate levels were shown to be an important factor for the recognition of severe TLS–AKI, as it can be considered a cost-effective laboratory marker of kidney involvement. A multidisciplinary approach is necessary to plan early preventive steps, such as optimization of hydration, application of adjuvant allopurinol and rUO treatment, and avoiding nephrotoxic drugs as much as possible. KRT remained an effective treatment modality of the most severe forms of TLS–AKI.

This could be the basis for further studies on the relationship between phosphate level and TLS. The major limitation of our study is the low number of patients, which may have a distorting effect on the results. Further studies with additional sensitive biomarkers and long-term follow-up data are needed for early detection and optimal management of severe TLS–AKI.

## Supplementary Information

Graphical Abstract (PPTX 65 KB)

## Data Availability

Datasets analyzed during the study are patients’ data available in their medical documentation and the patients’ electronic database (MedSolution).

## Abbreviations

ALL: Acute lymphoid leukemia
AKI: Acute kidney injury
Ca: Calcium
Cr: Creatinine
CTLS: Clinical tumor lysis syndrome
FE: Fractional excretion
F-pRIFLE: Failure category according to pRIFLe classification
GFR: Glomerular filtration rate
HPOG: Hungarian Pediatric Oncology Group
I-pRIFLE: Injury category according to pRIFLE classification
K: Potassium
KRT: Kidney replacement therapy
LDH: Lactate dehydrogenase
LTLS: Laboratory tumor lysis syndrome
Na: Sodium
NGAL: Neutrophil gelatinase-associated lipocalin
R-pRIFLE: Risk category according to pRIFLe classification
RAI: Renal angina index
rUO: Recombinant urate-oxidase
se: Serum
TLS: Tumor lysis syndrome
TmPO4/GFR: Ratio of maximum rate of renal tubular reabsorption of phosphate to GFR
UA: Uric acid
u: Urine
WBC: White blood cell
