# Supplementary material for: Daily serum phosphate increase as early and reliable indicator of kidney injury in children with leukemia and lymphoma developing tumor lysis syndrome
Source: Pediatr Nephrol. 2023 Mar 21;38(9):3117–27. doi: 10.1007/s00467-023-05923-z (PMC10432329; doi:10.1007/s00467-023-05923-z)
Supplement: Supplementary file 1 — Graphical Abstract (PPTX 65 KB) [file 467_2023_5923_MOESM1_ESM.pptx]

## Slide 1
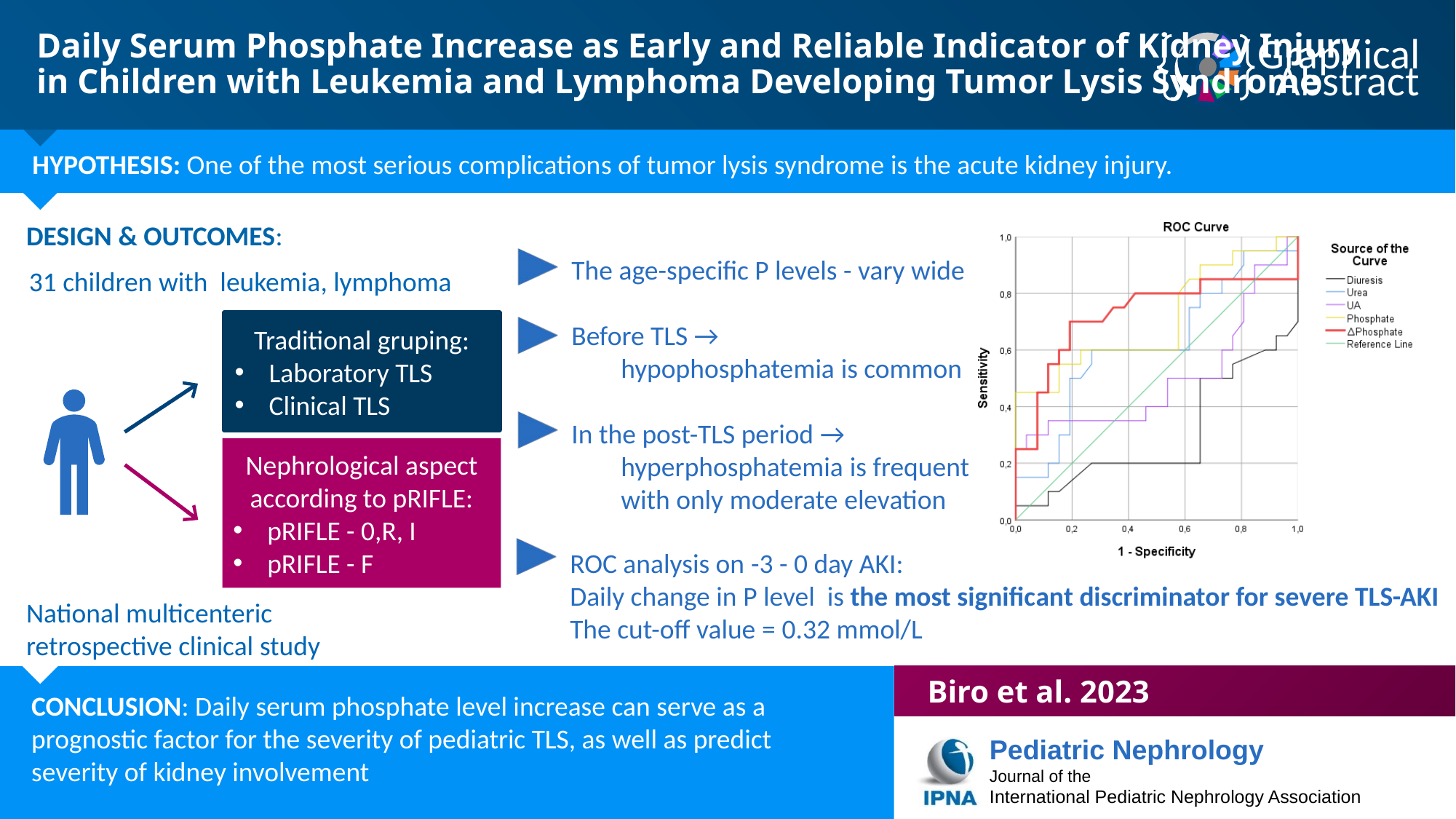

Daily Serum Phosphate Increase as Early and Reliable Indicator of Kidney Injury
in Children with Leukemia and Lymphoma Developing Tumor Lysis Syndrome
HYPOTHESIS: One of the most serious complications of tumor lysis syndrome is the acute kidney injury.
DESIGN & OUTCOMES:
The age-specific P levels - vary wide
Before TLS →
 hypophosphatemia is common
In the post-TLS period →
 hyperphosphatemia is frequent
 with only moderate elevation
31 children with leukemia, lymphoma
Traditional gruping:
Laboratory TLS
Clinical TLS
Nephrological aspect according to pRIFLE:
pRIFLE - 0,R, I
pRIFLE - F
ROC analysis on -3 - 0 day AKI:
Daily change in P level is the most significant discriminator for severe TLS-AKI The cut-off value = 0.32 mmol/L
National multicenteric
retrospective clinical study
Biro et al. 2023
CONCLUSION: Daily serum phosphate level increase can serve as a prognostic factor for the severity of pediatric TLS, as well as predict severity of kidney involvement
